# Reading and writing genomes

**DOI:** 10.1002/ggn2.10016

**Published:** 2019-07-29

**Authors:** Myles Axton, Alison Liu

**Affiliations:** ^1^ Wiley Hoboken New Jersey

1

Research in genetics provides the basis for understanding the function and evolution of all living things. The disciplines of reading and writing genomes translate into sustainable economic development with the rational global goals of food security, maternal and child health, precision medicine, education and access to informatics technologies. We believe that many publications in our field are motivated by these goals and contain reusable modular elements that can be recombined both in research and in its translation, to attain them. Open research entails sharing not only the conclusions of science, but its materials, provenance, and gestation for the widest reuse by human and computational users. This means that we and our readers deplore any hiding or obscuring data sets or methods, and regret data sets in formally public repositories that have very slow accession or transfer rates. However, we will endeavor to work with all data producers who make contributions in good faith to genetics and genomics research.

*Advanced Genetics* is an Open Research journal from Wiley, published online using the CC‐BY 4.0 open attribution license to encourage maximum credit and rapid creative reuse of all scholarly work. We are delighted to receive Original Research Articles, Resources, Analysis, Technical Reports, and Perspectives in the areas of human, animal, plant and microbial genetics, genomics, and epigenomics, selecting those reports for peer review that we judge editorially to have the highest research utility, ethical standards, and societal impact. As professional, full‐time editors at Wiley, we take responsibility for all manuscript decisions and peer reviewer assignment. Our Advisory Board Members have a complementary role to guide *Advanced Genetics's* mission as they see fit, anticipating the evolution of research and standards in our field, and, with us, providing leadership in promoting excellence in open research. Unlike Editorial Board members at some journals, *Advanced Genetics* advisors are our mentors, not manuscript editors. We welcome their commitment to the journal for as long as they wish, and advisors may leave or rejoin the board at will.

Since we offer an online journal, we are happy to consider reports in any format for peer review, provided they would not burden referees with their unusual length or complexity. We also welcome presubmission enquiries via our online database. Author and data set contributions and consortium roles can be described via the CRediT contributor taxonomy (https://www.casrai.org/credit.html). We support a range of community standards and databases and the FAIRSharing^1^ community standards site (https://fairsharing.org) for best practices and semantic precision. The journal endorses the FAIR^2^ data principles (https://www.go-fair.org/fair-principles/) and we recommend database submission of data sets and workflows to replace most of the prior use cases for Supplementary Information.

Research Articles should offer a new and substantial conceptual advance based on original experimental research and data, whereas Technical Reports need only detail a useful new method. Perspectives are literature reviews that set standards or propose future strategies in our field. Analysis articles offer opportunity to generate and test new hypotheses by interoperating or reusing existing data sets with new workflows. Resources provide provenance and curation of new data sets that will be of use to the community. If submissions are outside the scope of the journal or if editors consider them premature with respect to their field, we will make customized recommendation for appropriate Wiley journals that would peer review the work or suggest revisions that would typically qualify the work for peer review.

Enabling the market for genomics‐based ideas needs generosity with rich metadata and careful attention to semantic precision, as well as a sensitive understanding of the legal, ethical and economic underpinning of resources based in the code and the families of living people. For an editor, this means having patience in the face of the many exceptions to the ideal of publicly funded, universal research access to all human, animal, and plant genomes and their associated traits and measurements. The resource‐benefit balance is ever‐present, and legal and ethics frameworks of genetic research evolve slowly in the legacy of past abuses of concepts of heredity. It is therefore essential that we recognize those data license conditions that aim to preserve participation of research subjects, build local resources and capacity and return benefits to the societies that initiated the studies. So, when genetics advances only on the terms of a commercial animal breeder or a security‐conscious government, the conclusions and resources offered in the publication need to be maximized for reuse without derailing the sustainable long‐term commitment of those producers to make their results available. Even in the sphere of publicly funded data resources in developed countries, it may be networks of excellence (consortia) spanning continents, institutions, and generations of diverse funding sources that are the guarantors of the security of the research subjects' data and the translational success of the research. Publishers looking for a highly cited paper—or data reusers looking to test their new algorithm—need to see where they fit in, and lobby for greater FAIRness from well‐funded data generators. Proof of the reuse and interoperability of open research rests with the data users, so data providers need to enable and encourage their work.
